# Multi-Physics Monotone Score Transport for Unsupervised Domain Adaptation of Continuous Tool Wear Prediction

**DOI:** 10.3390/s26123873

**Published:** 2026-06-18

**Authors:** Enhao Cui, Runshan Hu, Weina Zhang, Zihan Fei, Chenyang Zhu

**Affiliations:** 1 School of Computer Science and Artificial Intelligence, Changzhou University, Changzhou 213164, China; 2500160612@smail.cczu.edu.cn (E.C.);; 2 School of Computer Engineering, Jimei University, Xiamen 361021, China; 3 Department of Electronics and Computer Science, University of Southampton, Southampton SO17 1BJ, UK

**Keywords:** tool wear prediction, unsupervised domain adaptation, intelligent manufacturing, regression adaptation

## Abstract

Cross-material continuous tool wear prediction is difficult because a model must preserve the physical wear scale, not only align high-dimensional sensor features. This limitation is critical in milling, where the target variable is the continuous flank wear width (VB) and material shift can distort the mapping from sensor response to wear magnitude. We address this problem by recasting cross-domain tool wear prediction as monotone wear-scale adaptation. We propose Multi-Physics Monotone Score Transport (MPMST), a monotone score transport framework that constructs a tool-wear-oriented score from sensor-derived candidate cues, transports the target-domain score onto the source-domain wear scale, and then predicts wear through isotonic regression. We also evaluate One-Physics Monotone Score Transport (OPMST), a force-only variant that uses the same score-transport pipeline with a restricted cue family. On Mondragon Unibertsitatea–Tool Condition Monitoring (MU-TCM) with two cross-material transfer tasks, the validation-driven MPMST configuration reduces mean absolute error by approximately 63% relative to Correlation Alignment (CORAL) and by approximately 31% relative to a physics-informed Gaussian process baseline. The results support monotone score construction and score transport as practical mechanisms for continuous tool wear prediction under domain shift, while also showing that MU-TCM is strongly force dominated.

## 1. Introduction

Continuous tool wear prediction is central to intelligent machining because flank wear directly affects dimensional accuracy, surface quality, cutting stability, and the usable life of expensive tooling. A model that estimates wear reliably across materials can therefore support both adaptive process control and economical tool replacement. In industrial deployment, however, a wear predictor trained on one material or cutting domain often degrades when applied to another, even when the sensing stack remains fixed. This cross-domain gap is especially severe in small-sample industrial settings, where collecting dense wear labels is costly and destructive. More importantly, the deployment failure is not merely a mismatch in source and target distributions; it is a mismatch in the mapping from sensed response to physical wear magnitude.

This deployment bottleneck points to a methodological gap. The broader transfer learning literature has long emphasized that transfer assumptions must match the target task structure [1]. Many unsupervised domain adaptation (UDA) pipelines are still centered on classification-style feature invariance [2]. Classical latent-subspace transfer methods such as Transfer Component Analysis (TCA) exemplify this viewpoint [3]. Covariance-alignment methods such as CORAL preserve a similar global-distribution philosophy [4]. Adversarial feature alignment later pushed the same agenda into deep representation learning [5]. Continuous tool wear prediction does not fit this regime cleanly. The target quantity is the continuous flank wear width (VB), and its evolution is fundamentally monotone. The relevant inductive bias is therefore not only domain invariance but also preservation of degradation order and wear scale. A method can reduce global domain discrepancy while still perturbing the continuous mapping from sensor response to physical wear, which is the failure mode of greatest concern in deployment.

We therefore adapt a low-dimensional, physically meaningful degradation coordinate instead of the full sensor distribution. MPMST implements this idea as a multi-physics monotone score transport framework for unsupervised domain adaptation of continuous tool wear prediction. The method constructs tool-wear-oriented cues from force, vibration, and acoustic-emission statistics, aggregates positively monotone cues into a one-dimensional wear score, and transports the target-domain score to the source wear scale before monotone score-to-VB prediction. The adaptation problem is thereby recast from high-dimensional feature matching into order-preserving wear-scale recovery. The contributions of this work are fivefold:

- We formulate unsupervised domain adaptation for continuous tool wear prediction as a monotone wear-scale adaptation problem rather than a class-alignment problem.
- We propose MPMST, which constructs a tool-wear-oriented multi-physics monotone score instead of adapting the full high-dimensional sensor distribution.
- We introduce cross-domain monotone score transport to recover the target-domain wear scale before isotonic continuous VB prediction.
- We introduce OPMST, a force-only single-physics variant with the same score-transport pipeline, to separate the contribution of the cue pool from the contribution of score transport.
- We validate the method on MU-TCM under cross-material transfer, where MPMST improves continuous tool wear prediction over classical UDA and recent physics-informed baselines in the main comparison.

## 2. Related Work

### 2.1. Unsupervised Domain Adaptation

UDA aims to transfer predictive models from a labeled source domain to an unlabeled target domain. Theoretical support for this setting is commonly traced to domain-divergence bounds developed by Ben-David et al. [6]. Early survey work positioned domain adaptation as one branch of the broader transfer learning landscape [1]. Broader transfer learning surveys also highlighted the role of task mismatch and negative transfer in practical deployment [7]. Later surveys documented how deep models changed the practical design space of adaptation methods [2]. Visual domain adaptation has also been reviewed from the perspective of transferable representation learning [8]. As a result, much of the UDA literature treats representation geometry as the main object to be aligned.

Classical UDA methods mainly align domains through global distribution matching. TCA does so through a shared latent subspace [3]. CORAL does so through covariance matching [4]. Deep Adaptation Networks extend discrepancy minimization into deep architectures through multi-kernel matching [9]. These approaches are attractive when a single invariant feature geometry is expected to explain both domains. Their limitation for tool wear is not that alignment is unnecessary, but that global feature matching does not explicitly preserve the scale of a continuous degradation variable.

Adversarial and classifier-based methods instead shape target features relative to source discriminative structure. Domain-Adversarial Neural Network (DANN) popularized adversarial domain confusion for representation learning [5]. Adversarial Discriminative Domain Adaptation (ADDA) relaxed shared-weight assumptions by learning an explicit target encoder [10]. Maximum Classifier Discrepancy (MCD) aligned target samples by minimizing classifier disagreement near decision boundaries [11]. Conditional Domain Adversarial Network (CDAN) conditioned adversarial alignment on classifier predictions [12]. Deep CORAL brought correlation alignment directly into end-to-end deep optimization [13]. These methods are effective when class boundaries are the central object to preserve, but they still leave open how to maintain the order and scale of a continuous health variable such as tool wear.

### 2.2. Continuous, Monotone, and Physics-Informed Prediction

Compared with classification UDA, continuous prediction under shift is less explored and often more fragile because misalignment directly perturbs the target scale. Monotone regression offers one principled response when the target variable evolves cumulatively. Isotone optimization provides a classical route to order-preserving regression [14]. Shape-constrained additive models show that monotonicity can also be enforced in more flexible nonlinear predictors [15]. These methods are directly relevant because tool wear is not only continuous but also ordered in time and severity. However, they do not by themselves solve the cross-domain calibration problem.

Physics-informed learning addresses a related problem from the side of model bias. Physics-informed neural networks demonstrated that known structure can regularize continuous prediction even under sparse supervision [16]. A broader review of physics-informed machine learning highlighted this idea as a general strategy for scientific and engineering inference [17]. In tool wear prediction, Zhu et al. integrated physics priors into Gaussian process regression for continuous wear estimation [18]. Kim et al. later combined physics guidance with unsupervised adaptation and prediction adjustment across operating conditions [19]. Our method differs by placing the physical prior on a monotone score that is explicitly transported across domains.

### 2.3. Tool Wear Monitoring and Prediction

Tool wear monitoring has a long history in indirect sensing and process diagnostics. State-of-the-art reviews established force, vibration, and acoustic emission as the dominant sensing channels in machining [20]. Flank wear prediction in turning was later reviewed from the perspective of sensing, feature extraction, and decision making [21]. Milling-focused surveys emphasized the additional complexity induced by intermittent cutting and variable cutter engagement [22]. Later reviews summarized how machine learning entered milling-oriented monitoring pipelines [23]. Adaptive monitoring surveys stressed the need for robust online updating in production environments [24]. Integrated monitoring-system reviews expanded the scope from isolated algorithms to deployable sensing-and-decision stacks [25]. A review devoted to turning highlighted how indirect multi-sensor systems remained difficult to generalize across setups [26]. High-performance machining reviews then pushed the discussion toward robustness, dimensionality reduction, and wireless sensing readiness [27]. Recent keynote work on process monitoring in machining reinforced the importance of practical sensor integration for industrial adoption [28]. A deployable wear predictor therefore needs both sensor-aware feature construction and domain-aware calibration.

Recent learning-based tool wear models increasingly target robustness across conditions, but they usually do so without an explicit continuous adaptation formulation. The MU-TCM dataset provides a compact benchmark for studying this issue under cross-material transfer [29]. Wang et al. proposed a sensing-generalization strategy for milling wear monitoring [30]. Zhang et al. combined hybrid sensor information with deep transfer learning for wear monitoring [31]. He et al. explored adversarial data augmentation for transfer-oriented tool wear prediction [32]. Hassan et al. proposed a multi-stage deep learning framework for milling wear-level prediction [33]. Related industrial UDA work has also used statistically aligned feature augmentation to improve robustness in fault diagnosis under heterogeneous operating conditions [34]. These studies broaden the operating conditions, sensor settings, and transfer scenarios considered in tool monitoring, but they do not explicitly formulate continuous cross-domain tool wear prediction as monotone score adaptation.

### 2.4. Physics-Informed and Monotone Modeling

Physics-guided learning uses known structure to regularize data-driven models. Multi-sensor monitoring has long been recognized as a prerequisite for reliable machining diagnostics [35]. Outside machining, multi-modal robotic perception studies also show the value of balancing complementary sensing channels under illumination and modality changes [36]. Force-informed Gaussian processes provide one example of physically biased continuous wear modeling [18]. FEM-assisted subdomain adaptation shows how simulation can supply cross-domain structure for monitoring models [37]. Physics-guided prediction adjustment extends the same intuition to novel operating conditions [19]. Here, the physical prior is encoded as a tool-wear-specific monotone score built from multi-physics cues and adapted by one-dimensional transport rather than full feature alignment. Compared with prior work, this formulation explicitly combines multi-physics cue construction, monotone score aggregation, and score-space transport for unsupervised domain adaptation of continuous tool wear prediction.

## 3. Problem Formulation

We consider unsupervised domain adaptation for continuous tool wear prediction. The labeled source domain is $D_{s}={\left\{(x_{i}^{s},y_{i}^{s})\right\}}_{i=1}^{n_{s}}$, where $x_{i}^{s}\in R^{d}$ is a multi-sensor feature vector, and $y_{i}^{s}\in R_{\geq 0}$ is the continuous flank wear width (VB). The target domain is $D_{t}={\left\{x_{j}^{t}\right\}}_{j=1}^{n_{t}}$, where target-domain wear labels are unavailable during adaptation. In MU-TCM, the source and target domains correspond to different workpiece materials, leading to cross-material shifts in the sensor statistics.

The objective is to learn a predictor $f:R^{d}\to R_{\geq 0}$ that accurately estimates target-domain continuous tool wear as ${\hat{y}}_{j}^{t}=f\left(x_{j}^{t}\right)$ using labeled source data and unlabeled target data only.

Although binary wear classification and ordinal wear staging are useful for reporting, they are secondary views of the problem. The primary task is continuous VB prediction: a model can preserve thresholded labels while still distorting the continuous wear scale.

Our key assumption is that there exists a latent, physically meaningful monotone tool wear coordinate s(x) such that tool wear increases monotonically with the coordinate within each domain. Domain shift then acts mainly by distorting the distribution and scale of this coordinate rather than destroying its order. Under this view, adapting s(x) is expected to be more stable than aligning the full high-dimensional sensor distribution. The problem is therefore formulated as unsupervised domain adaptation for continuous tool wear prediction, with both the method and the experimental design organized around this score-space formulation.

## 4. The MPMST Framework for Monotone Wear-Scale Adaptation

**Figure 1:**
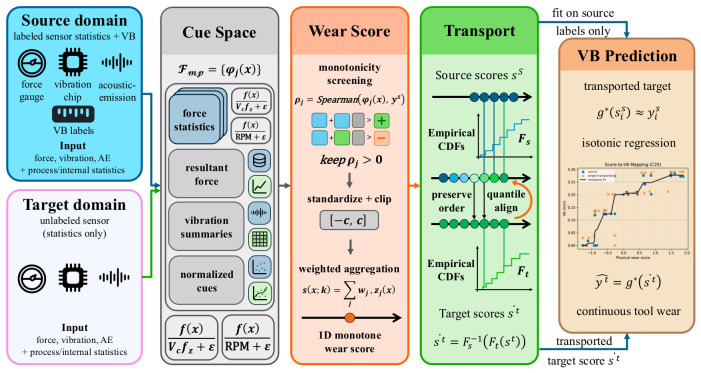
Overview of MPMST. The framework receives labeled source-domain sensor statistics and unlabeled target-domain sensor statistics, constructs a multi-physics cue space, compresses the cues into a monotone wear score, transports the target score to the source wear scale by one-dimensional quantile alignment, and finally predicts continuous target-domain flank wear by a source-fitted isotonic regressor.

MPMST adapts continuous tool wear prediction by constructing a one-dimensional tool wear score from multi-physics sensor cues, transporting the target-domain score to the source wear scale, and then applying a monotone score-to-VB predictor. Figure 1 summarizes the pipeline. Module I handles raw sensor evidence under material shift. Module II replaces high-dimensional feature alignment with a monotone wear coordinate. Module III restores cross-domain comparability in the continuous wear scale.

### 4.1. Constructing Tool-Wear-Oriented Multi-Physics Cues

The first module separates tool-wear-relevant variation from raw domain-specific scale effects before adaptation. Let $x\in R^{d}$ denote a vector of sensor statistics computed from force, vibration, acoustic-emission, and machine-internal process signals when available. We form a candidate cue pool $F_{\mathrm{mp}}={\left\{\phi_{j}\left(x\right)\right\}}_{j=1}^{D}$ whose elements include both original statistics and derived force-load normalized quantities. The pool is organized to expose wear-sensitive responses that are less tied to raw operating scale. In the present implementation, $F_{\mathrm{mp}}$ combines force-axis statistics, resultant-force magnitudes, force-axis ratios, vibration candidates, acoustic-emission candidates, computer numerical control (CNC)-recorded internal process candidates, and load-normalized force descriptors.

In particular, if f(x) denotes a force-related magnitude, and $\left(V_{c},\;f_{z},\mathrm{RPM}\right)$ are cutting-condition variables, we construct normalized cues according to ${\tilde{f}}_{V_{c}f_{z}}\left(x\right)=\frac{f(x)}{V_{c}f_{z}+\epsilon }$ and ${\tilde{f}}_{\mathrm{RPM}}\left(x\right)=\frac{f(x)}{\mathrm{RPM}+\epsilon }$ with a small ϵ>0 for numerical stability. This inline normalization pattern is used for resultant-force summaries and selected acoustic-emission descriptors when the corresponding operating-condition variables are available.

The module exposes descriptors that respond to wear progression in complementary ways: force growth captures cutting resistance, vibration statistics reflect dynamic instability, and acoustic-emission energy reflects high-frequency frictional activity. The normalized cues reduce the chance that the downstream score simply tracks cutting-condition scale rather than physical wear.

One-Physics Monotone Score Transport (OPMST) is the force-only single-physics variant of MPMST. It retains the same monotone score construction, cross-domain score transport, and isotonic wear prediction pipeline, but restricts the input cue family to force-dominated features, denoted $F_{\mathrm{force}}$. This force-only family is not a literal subset of the compact MPMST implementation pool; instead, OPMST uses a broader force descriptor set so that the single-physics baseline is not weakened by an overly narrow force representation. By contrast, MPMST uses a compact multi-physics candidate pool $F_{\mathrm{mp}}$ that includes selected force, vibration, acoustic-emission, and CNC-recorded internal process descriptors. The multi-physics designation therefore refers to the candidate cue pool before monotonicity screening, not to a guarantee that every sensing modality will enter the final score on every dataset.

### 4.2. Learning a Monotone Tool Wear Coordinate

The second module compresses the cue pool into a one-dimensional monotone tool wear score. This coordinate replaces a high-dimensional representation with a variable explicitly aligned with wear progression. Statistically, the compression reduces variance in the small-sample regime by replacing many weakly reliable dimensions with a single calibrated coordinate. Physically, it retains cues that evolve in the same direction as accumulated flank wear.

The first step is to discard cues whose direction of change is inconsistent with accumulated wear. For each candidate cue $\phi_{j}$, we compute its source-domain monotonicity with respect to the continuous wear label VB using the Spearman coefficient $\rho_{j}=\mathrm{Spearman}\left(\phi_{j}\left(x^{s}\right),y^{s}\right)$. We then retain only positively monotone cues:

(1) $$J_{+}=\left\{j:\rho_{j}>0\right\},$$

and rank them by decreasing $\rho_{j}$. Equation (1) gives the admissible set of cues whose variation is at least directionally compatible with wear growth. Cues with weak, unstable, or sign-inconsistent wear relations are removed because they are especially vulnerable to spurious cross-material variation.

After the monotone subset has been identified, the next step is to place the retained cues on a comparable numerical scale. For each selected cue, we compute the source-domain standardization

(2) $$z_{j}\left(x\right)=\mathrm{clip}\;\left(\frac{\phi_{j}\left(x\right)-\mu_{j}^{s}}{\sigma_{j}^{s}+\delta },-c,c\right),$$

where $\mu_{j}^{s}$ and $\sigma_{j}^{s}$ are the source statistics of cue *j*, δ>0 avoids numerical instability, and *c* is a clipping constant. Equation (2) defines the robust normalization that prevents a small number of high-variance cues from dominating the downstream score. Once all retained cues are standardized, the monotone tool wear score is formed as

(3) $$s\left(x;k\right)=\sum_{j\in J_{k}}w_{j}\;z_{j}\left(x\right),\;w_{j}=\frac{\rho_{j}}{\sum_{\ell \in J_{k}}\rho_{\ell }},$$

where $J_{k}\subset J_{+}$ contains the top-*k* positively monotone cues.

Equation (3) is the central construction of Module II: it turns a heterogeneous set of standardized cues into a single coordinate that increases with wear in expectation. This score is not a generic latent embedding. Its geometry is deliberately constrained by the top-*k* screening and the monotonicity-based weights, so it can be read as a soft consensus over wear-consistent physical evidence rather than as a black-box latent variable.

Because the score dimension controls the bias-variance trade-off of the whole method, it is selected explicitly rather than fixed heuristically. In implementation, the score dimension is chosen without target labels by minimizing source-validation error:

(4) $$k^{★}=\arg\min_{k\in K}\frac{1}{n_{\mathrm{val}}}\sum_{i=1}^{n_{\mathrm{val}}}\left|g_{k}\;\left(s(x_{i}^{s,\mathrm{val}};k)\right)-y_{i}^{s,\mathrm{val}}\right|,$$

where K is the candidate set of score dimensions, and $g_{k}$ is the monotone score-to-wear map fitted on the source training split for that value of *k*. Equation (4) specifies the source-only model-selection rule used in the implementation. In the experiments, we evaluate both uniform weighting and fixed-*k* alternatives to quantify the contribution of this module.

### 4.3. Transporting the Wear Coordinate Across Domains

The third module adapts the target-domain score to the source wear scale and predicts continuous tool wear. It preserves target-domain order while restoring comparability of wear scale across domains. Once Module II has produced a one-dimensional score, the key remaining mismatch is not feature geometry but wear-scale calibration.

To adapt the score across domains, we first need a compact description of the wear-scale geometry in each domain. Let $s^{s}$ and $s^{t}$ denote the source and target scores. Their empirical cumulative distribution functions are

(5) $$F_{d}\left(u\right)=\frac{1}{n_{d}}\sum_{i=1}^{n_{d}}1\left[s_{i}^{d}\leq u\right],\;d\in \left\{s,t\right\},$$

where 1[·] is the indicator function. Equation (5) formalizes the cumulative ordering structure that the transport step will preserve. With these empirical geometries in hand, we transport each target score by one-dimensional quantile alignment:

(6) $${\tilde{s}}^{t}=F_{s}^{-1}\;\left(F_{t}\left(s^{t}\right)\right).$$

Equation (6) preserves target-domain ordering while re-scaling the score onto the source wear coordinate. The mapping is implemented from the empirical distributions in Equation (5) by sorted samples with linear interpolation between neighboring quantiles. The step is intentionally one-dimensional: once the score has been constructed, the adaptation problem becomes a wear-scale calibration problem rather than a full feature-geometry problem. The mapping is simpler than high-dimensional feature matching and also easier to regularize in the small-sample regime.

Once the transported score lies on the source wear scale, the remaining task is to convert that score into a continuous VB estimate. Given transported target scores, we learn a monotone score-to-wear predictor on the source domain through isotonic regression:

(7) $$g^{★}=\arg\min_{g\in G_{↑}}\sum_{i=1}^{n_{s}}{\left(g\left(s_{i}^{s}\right)-y_{i}^{s}\right)}^{2},$$

where $G_{↑}$ is the set of non-decreasing functions. Equation (7) defines the monotone calibration stage that fits wear magnitude without violating the assumed order structure. The final target-domain prediction is then

(8) $${\hat{y}}^{t}=g^{★}\left({\tilde{s}}^{t}\right).$$

Equation (8) closes the inference pipeline by composing transport with monotone calibration. The isotonic model preserves the assumed monotone relationship between score and wear without imposing an unnecessarily rigid parametric form on the final score-to-VB map. During model selection, the score dimension is chosen on a source-side validation split; after selection, the isotonic predictor is refit on all labeled source samples and then applied to transported target scores.

Equations (3)–(8) summarize the mechanism of the proposed framework. For continuous tool wear prediction, preserving the order and scale of a one-dimensional wear coordinate can be more stable than aligning a high-dimensional sensor space whose geometry may change with material.

The implementation of MPMST uses the multi-physics cue pool $F_{\mathrm{mp}}$, while OPMST shares the same score-transport-and-prediction machinery under the restricted force-only cue pool. Both variants are computationally lightweight on MU-TCM because all computations are one-dimensional after score construction.

## 5. Experiments

### 5.1. Experimental Setup

We evaluate MPMST on MU-TCM [29], a small but challenging tool wear benchmark for face milling across low- to high-yield-strength materials. The benchmark used here contains 67 samples and 228 original sensor statistics, with continuous flank wear width (VB) as the target variable. The two domains correspond to CastIron.GG30 (34 samples) and StainlessSteel.316L (33 samples). The evaluation focuses on continuous cross-material prediction accuracy, statistical reliability under small samples, the contribution of each method component, cue-selection stability, and diagnostics of the monotone-score assumption. Experimental protocol is provided in Table 1.

We define two cross-material transfer tasks:

1. CastIron→Stainless: train on labeled cast iron, adapt to unlabeled stainless steel.
2. Stainless→CastIron: train on labeled stainless steel, adapt to unlabeled cast iron.

Source labels are split into train/validation partitions for each random seed, while target labels are used only for evaluation. Continuous VB prediction is the primary objective. We additionally report binary F1 at VB≥0.2 mm and four-stage ordinal macro-F1 using thresholds at 0.05, 0.15, and 0.25 mm. These ordinal thresholds are reporting bins for stage-level consistency on MU-TCM rather than ISO wear-limit definitions.

Table 2 summarizes the cutting-condition range in the evaluated subset. Axial and radial depths of cut are fixed in MU-TCM, while cutting speed and feed per tooth vary by material. The force-load normalized cues in Section 4.1 should therefore be read as compact descriptors for this benchmark rather than as a claim of invariance under arbitrary cutting-condition changes.

The source-only comparison uses ridge regression [38], which provides a baseline for evaluating the need for adaptation. The classical adaptation baselines are CORAL [4] and TCA [3], which assess whether generic distribution alignment is sufficient in this setting. Physics-informed regression is represented by PI-GPR [18]. Physics-guided adaptation is represented by Finite Element Method-assisted Deep Subdomain Adaptation Network (FEM-DSAN) [37] and PG-UDA-PGA [19]. Because MU-TCM is a compact tabular benchmark and official implementations are not uniformly available, the recent deep baselines are reproduced as mechanism-preserving counterparts under the same protocol; the comparison therefore evaluates adaptation mechanisms rather than full-scale deep architectures.

All reported numbers are averaged over 50 random seeds with source-side train/validation splits determined by the seed. For each transfer direction, the labeled source domain is partitioned with a stratified validation split using the binary wear label at VB=0.2 mm, and the validation size is set to $\max(0.2,4/n_{s})$ of the source samples. Target labels are never used for model selection. Missing feature values are median-imputed on the source train split, then standardized with source-train statistics and clipped to [−8,8]. In Equation (2), we set $\delta =10^{-6}$ and c=8. Ridge-based baselines select the regularization coefficient from {0.01,0.1,1,10,100} using source-validation Mean Absolute Error (MAE). OPMST and MPMST select the score dimension from top-k∈{2,…,10} by source-validation MAE, refit on all labeled source samples, and use isotonic regression for the final score-to-VB mapping. We report standard deviations across seed–direction units, 95% bootstrap confidence intervals, and paired Wilcoxon signed-rank tests with Holm adjustment. For the overall tests, Holm adjustment is applied over all MPMST-versus-comparison metric tests in the main experiment; direction-wise tests are adjusted separately within each transfer direction.

### 5.2. Comparison with Existing Methods

Table 3 reports the main comparison for continuous cross-material tool wear prediction. The validation-driven MPMST configuration achieves the lowest overall MAE of 0.034, the lowest RMSE of 0.058, the highest $R^{2}$ of 0.669, and the highest ordinal macro-F1 of 0.786. Generic feature-alignment baselines are less effective in this continuous setting: MPMST reduces MAE by approximately 63% relative to CORAL and by approximately 31% relative to PI-GPR. The force-only variant OPMST remains a strong baseline, which confirms that force cues contain a substantial monotone wear signature. The MPMST–OPMST comparison should be read directionally rather than as a direction-agnostic multi-physics gain: the reliable margin appears on CastIron→Stainless, while the two variants are statistically indistinguishable on Stainless→CastIron.

Table 4 reports point estimates, 95% bootstrap confidence intervals (CIs), and Holm-adjusted paired-test results for the direct comparison between MPMST and OPMST. Across 100 paired seed–direction units, MPMST improves all five reported metrics after Holm adjustment. The direction-wise tests show that the statistically reliable margin comes mainly from CastIron→Stainless; for MAE, the Holm-adjusted paired-test value is $5.1\times 10^{-5}$ on CastIron→Stainless and 1.000 on Stainless→CastIron, and for ordinal macro-F1 it is $1.11\times 10^{-3}$ on CastIron→Stainless and 0.583 on Stainless→CastIron. These direction-wise values use the same Holm procedure but are adjusted separately within each transfer direction. This result narrows the claim: the multi-physics cue pool is useful in the direction where the MPMST–OPMST margin is clearer, while force-only cues already explain much of the wear ordering in MU-TCM. The CIs in Table 4 summarize marginal uncertainty across seed–direction units, whereas the paired Wilcoxon tests account for the matched seed–direction design.

Direction-wise results show where the advantage comes from. MPMST obtains its largest margin on CastIron→Stainless, where generic feature alignment is especially unstable, and it remains competitive on Stainless→CastIron. In contrast, CORAL and TCA vary substantially across directions, while PG-UDA-PGA loses much of its advantage on Stainless→CastIron. This behavior is consistent with the claim that score-level adaptation is preferable when the main deployment risk is wear-scale distortion rather than class confusion.

### 5.3. Ablation Study

The ablation study in Table 5 evaluates the contribution of the three modules introduced in Section 4. Module I is tested by replacing multi-physics cues with force-only cues. Module II is tested by perturbing the score-construction rule through fixed-*k* and uniform-weight variants. Module III is tested by removing score transport. The ablations are interpreted as diagnostics rather than as target-side model-selection rules; the default MPMST uses only source-validation information to choose *k*.

The ablations show that score transport is the most influential component of the framework: removing it degrades MPMST MAE from 0.034 to 0.063 and ordinal macro-F1 from 0.786 to 0.425, and removing it from OPMST produces a similar degradation. The force-only variants perform well, and the minimal force-pair diagnostic gives the lowest MAE and binary F1 among the Module I variants. Paired Wilcoxon tests over the 100 seed–direction units confirm that these differences are not merely descriptive: relative to default MPMST, the minimal force pair is significantly better on MAE (Holm-adjusted $p=1.02\times 10^{-3}$), RMSE ($p=1.04\times 10^{-4}$), $R^{2}$ ($p=1.31\times 10^{-4}$), and binary F1 ($p=2.24\times 10^{-9}$), while MPMST is significantly better on ordinal macro-F1 ($p=1.33\times 10^{-2}$). This result means that a multi-physics cue pool is not strictly necessary for low continuous-error metrics on MU-TCM. The minimal pair’s ordinal macro-F1 is lower than that of MPMST, however, which indicates that excellent threshold-level behavior does not necessarily preserve the full wear progression. The fixed top-k=6 diagnostic also slightly improves MAE, while the source-validation configuration remains the default because it avoids selecting a global *k* from target-side performance. Overall, the ablations support score transport as the central calibration mechanism and show that MU-TCM is strongly force dominated; the multi-physics pool should be interpreted as a broader calibration option rather than as a universally necessary ingredient on this benchmark.

### 5.4. Hyperparameter Sensitivity

**Figure 2:**
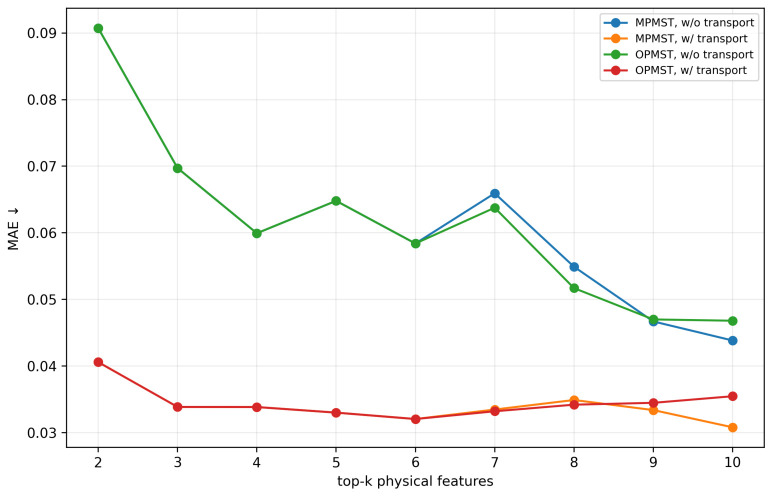
Sensitivity of target-domain mean absolute error to the number of selected monotone tool wear cues. The plotted curve is computed with target-domain labels after training for diagnostic analysis only; the default model selects *k* by source validation rather than target labels.

**Figure 3:**
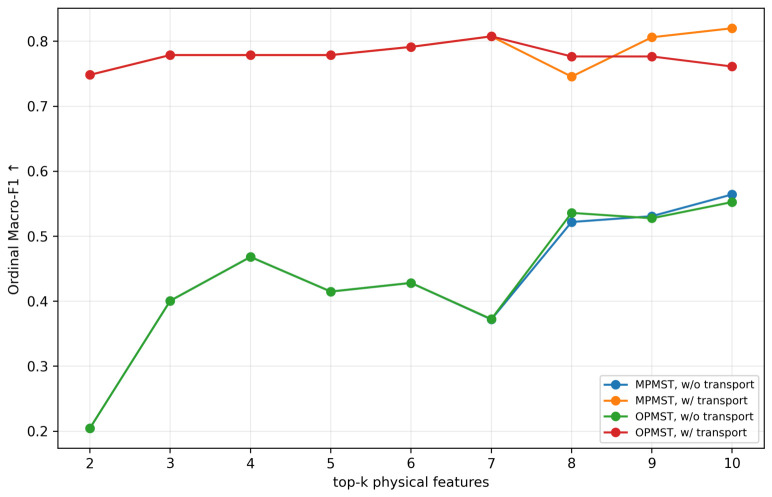
Sensitivity of ordinal macro-F1 to the number of selected monotone tool wear cues. The plotted curve is computed with target-domain labels after training for diagnostic analysis only. Score transport provides the dominant improvement, while larger cue counts can further improve the target-side diagnostic curve.

Figure 2 and Figure 3 report sensitivity to the number of selected monotone cues. Figure 2 evaluates continuous prediction error, while Figure 3 evaluates ordinal stage consistency. The sweep uses target labels only for analysis after training; it is not used to choose the default model. With score transport enabled, MPMST maintains a low-error region over a wide range of *k*, and the target-side sweep reaches its best MAE at a larger cue count. Without score transport, both MPMST and OPMST degrade substantially, confirming that cue selection alone is not sufficient for cross-material scale recovery.

Module II is not tied to a single cue count, but cue-count selection remains a real source of variance in this small benchmark. Across 50 seeds per direction, the source-validation rule selected an average top-*k* of 7.04 for CastIron→Stainless and 4.08 for Stainless→CastIron in MPMST. The diagnostic sweep shows that fixed larger values can improve target-side performance, but using those values as the default would amount to post hoc target tuning. We therefore keep the validation-driven configuration as the main protocol and report fixed-*k* variants only as ablations.

### 5.5. Score Assumption and Cue-Stability Diagnostics

The monotone-score hypothesis is empirical rather than guaranteed. To test whether the learned score preserves a usable target-domain wear ordering, we computed target-domain Spearman correlation, Kendall-style pairwise order-violation rates, raw source–target score-range coverage, source–target score-distribution discrepancy, and cue-set stability across seeds. Table 6 summarizes the results for the default MPMST and the force-only OPMST variant. The candidate cue-pool column reports the predefined candidate pool before positive Spearman screening and top-*k* selection.

Both scores are strongly but not perfectly monotone in the target domain. The approximately 9–10% pairwise order-violation rate means that quantile transport should be understood as a calibrated approximation, not as a formal guarantee that material shift preserves all wear ranks. The raw range coverage and KS values indicate that source and target scores remain shifted before transport. Cue-selection stability is moderate rather than complete. The selected MPMST cues are force dominated: across the 50-seed-per-direction evaluation, 536 selected cue instances are force cues, 20 are CNC-recorded internal process cues, and 0 are vibration or acoustic-emission cues. Thus, the vibration and AE descriptors are part of the candidate pool but are not selected by the source-validation top-*k* rule on MU-TCM. Although the selected cues are mostly force descriptors, the MPMST force descriptors are drawn from a different compact curated pool than the broader OPMST force-only set, and the occasional CNC-recorded internal process selections contribute to the observed MPMST–OPMST margin on CastIron→Stainless. This supports the revised interpretation that MPMST offers a broader cue pool for score construction, while the evidence in this benchmark is driven mainly by force cues and score transport.

### 5.6. Qualitative Analysis

**Figure 4:**
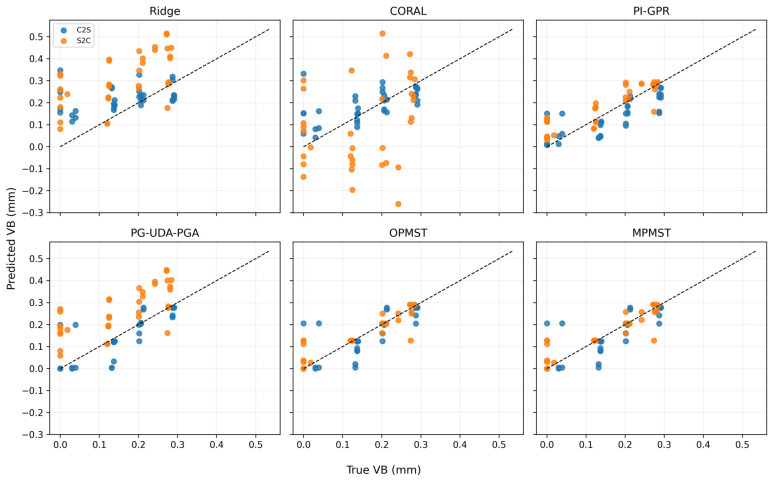
Predicted-versus-true continuous tool wear across representative baselines and the proposed method. The scatter concentration around the diagonal indicates that MPMST recovers the target wear scale more faithfully than generic alignment or source-only regression.

**Figure 5:**
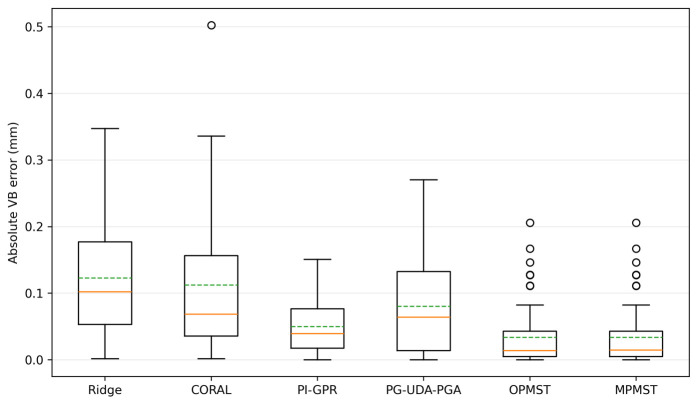
Absolute-error distribution across methods. The narrower and lower error profile of MPMST indicates that the gain is distributed across samples rather than driven by a small number of easy target points. Orange line for median absolute error and green line for mean absolute error accross all samples.

Figure 4 compares predicted-versus-true wear trajectories across methods. The predictions of MPMST are more concentrated around the diagonal than those of CORAL, ridge regression, or the recent physics-guided baselines. Figure 5 shows that the improvement is distributed across samples rather than driven by a small number of outliers. The more compact error distribution is consistent with improved wear-scale calibration.

**Figure 6:**
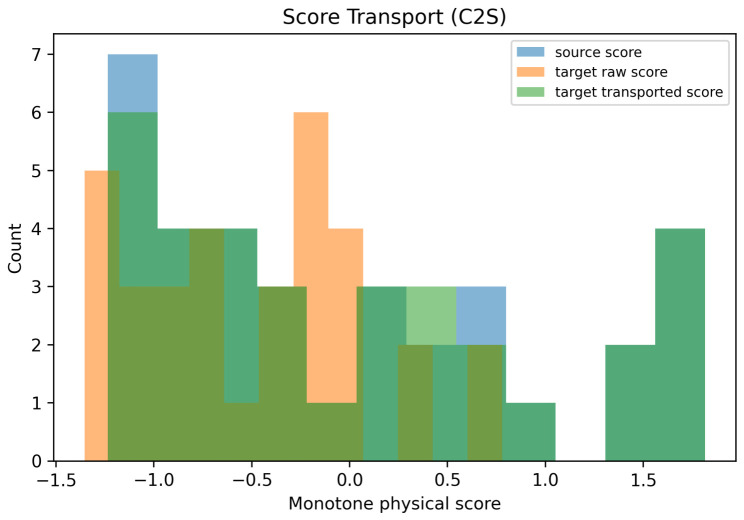
Distribution of source scores, raw target scores, and transported target scores on the CastIron→Stainless transfer. Quantile transport reduces the cross-domain wear-scale mismatch while preserving target ordering.

**Figure 7:**
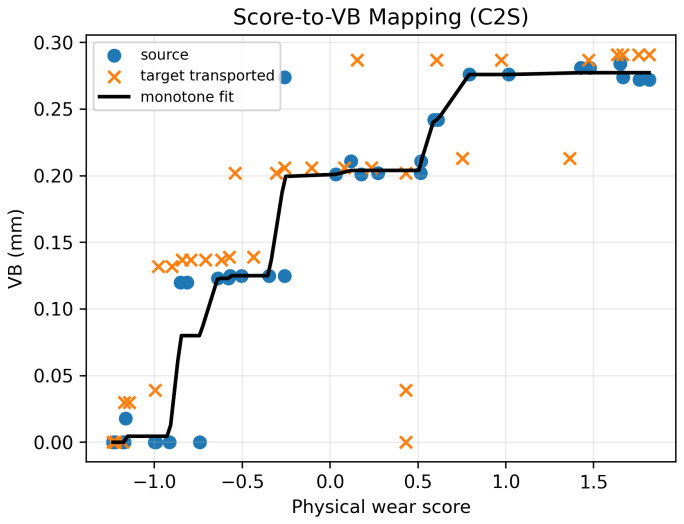
Monotone score-to-VB mapping on the CastIron→Stainless transfer. The fitted isotonic curve calibrates transported target scores on the source wear scale and converts them into continuous tool wear predictions.

Figure 6 links the improvement to the mechanism of the method. The raw target score distribution is shifted relative to the source, but quantile transport restores a comparable scale before isotonic score-to-VB prediction. Figure 7 shows how the transported target score is interpreted by the monotone score-to-wear map.

The qualitative results support the score-space calibration design while also showing the strength of force cues in MU-TCM. The force-only variants perform well near simple decision thresholds, but MPMST gives a stronger overall balance between continuous accuracy and ordinal consistency in the main validation-driven protocol. The evidence therefore supports a calibrated monotone score as the main mechanism and a broader cue pool as a useful, but dataset-dependent, extension. The gain from MPMST is mainly a calibration gain: it restores a more coherent wear scale rather than only correcting isolated samples.

## 6. Discussion and Limitations

The comparison with recent deep and physics-guided methods is intended as a controlled mechanism comparison under a compact tabular protocol. Because official implementations and full training recipes are not uniformly available for all recent tool-wear adaptation models, FEM-DSAN and PG-UDA-PGA are reproduced as mechanism-preserving counterparts with common source-validation selection. This design avoids giving deep models target labels or additional data.

Finally, MPMST requires labeled source wear measurements, source-domain statistics, and enough overlap in wear coverage for target score transport to be meaningful. The condition-stratified results are mixed: MPMST is statistically better than OPMST on the CastIron→Stainless transfer, with a Holm-adjusted *p* value of $5.1\times 10^{-5}$ for MAE, but statistically indistinguishable from OPMST on Stainless→CastIron. This behavior reinforces the revised claim that MPMST is a lightweight score-space adaptation framework whose multi-physics cue pool can improve calibration only when additional sensing channels survive the monotone-screening and validation process, while force cues remain highly informative in the evaluated benchmark.

## 7. Conclusions

This study addressed unsupervised domain adaptation for continuous tool wear prediction under cross-material shift. The proposed formulation treats continuous tool wear UDA as monotone wear-scale adaptation rather than class alignment. MPMST implements this formulation by constructing a tool-wear-oriented monotone score from a multi-physics candidate pool, transporting that score across domains, and applying isotonic continuous VB prediction on the transported scale. Experiments on MU-TCM show that these design choices improve overall continuous prediction accuracy and ordinal consistency relative to the evaluated cross-domain baselines under the reported protocol. The strongest evidence is for score transport as a calibration step. The additional benefit of the multi-physics candidate pool over the force-only OPMST variant is direction-conditional: it is statistically reliable on CastIron→Stainless but absent on Stainless→CastIron in the present benchmark.

Future work should focus on reliability mechanisms for score-space adaptation, including stability-aware cue selection, diagnostics for rank-order violations, and fallback calibration when the transported score is unreliable. The formulation may also apply to other cumulative industrial health variables, but the monotone-score assumption and the role of each cue family should be verified for each new degradation process.

## Figures and Tables

**Table 1:** Experimental protocol on MU-TCM.

Item	Value
Target variable	Continuous flank wear width (VB)
Sensor representation	228 statistics + derived multi-physics cues
Domains	Cast iron and stainless steel
Transfer tasks	CastIron→Stainless, Stainless→CastIron
Adaptation setting	Labeled source, unlabeled target
Primary metric	MAE on continuous VB
Secondary metrics	RMSE, $R^{2}$, binary F1, ordinal macro-F1; Spearman diagnostics are reported in Table 6

**Table 2:** Cutting-condition range and wear coverage in the evaluated MU-TCM subset.

Material	$V_{c}$	$f_{z}$	$a_{p}$	$a_{e}$	Samples	VB Range
CastIron.GG30	{100, 200}	{0.10, 0.20}	1.5	58.4	34	0.000–0.284
StainlessSteel.316L	{50, 100}	{0.05, 0.10}	1.5	58.4	33	0.000–0.291

**Table 3:** Main comparison for cross-material continuous tool wear prediction over 50 random seeds. MAE is the primary metric. Best values are highlighted in bold according to unrounded means. Table entries are rounded to three decimals; relative reductions discussed in the text use unrounded seed–direction means.

Method	CastIron→Stainless MAE	Stainless→CastIron MAE	Overall MAE	RMSE	$R^{2}$	Binary F1 (VB ≥ 0.2 mm)	Ordinal Macro-F1
Ridge [38]	0.086	0.125	0.106	0.129	−0.618	0.769	0.247
CORAL [4]	0.103	0.081	0.092	0.115	−0.573	0.696	0.343
TCA [3]	0.087	0.091	0.089	0.108	−0.148	0.481	0.264
FEM-DSAN [37]	0.097	0.100	0.098	0.125	−0.534	0.668	0.317
PI-GPR [18]	0.054	0.046	0.050	0.063	0.610	0.805	0.537
PG-UDA-PGA [19]	0.048	0.098	0.073	0.094	0.064	0.863	0.494
OPMST	0.042	**0.029**	0.035	0.059	0.652	0.885	0.772
MPMST	**0.040**	0.029	**0.034**	**0.058**	**0.669**	**0.892**	**0.786**

**Table 4:** Uncertainty estimates and paired tests for MPMST and the force-only OPMST variant. Values are reported as mean (95% bootstrap CI).

Metric	MPMST Mean (95% CI)	OPMST Mean (95% CI)	Holm-Adjusted *p* Value
MAE	0.034 (0.033–0.036)	0.035 (0.034–0.037)	$1.89\times 10^{-4}$
RMSE	0.058 (0.056–0.060)	0.059 (0.057–0.061)	$1.29\times 10^{-3}$
$R^{2}$	0.669 (0.648–0.690)	0.652 (0.630–0.675)	$7.66\times 10^{-4}$
Binary F1 (VB ≥ 0.2 mm)	0.892 (0.885–0.900)	0.885 (0.877–0.893)	$2.66\times 10^{-3}$
Ordinal macro-F1	0.786 (0.779–0.793)	0.772 (0.764–0.780)	$4.90\times 10^{-3}$

**Table 5:** Module-wise ablation of MPMST over 50 random seeds. Within each module block, bold marks the best unrounded value for each metric. The fixed-*k* and minimal-force variants are diagnostic configurations; the default MPMST selects *k* by source validation. The default OPMST row is repeated in Module III to make the transport/no-transport contrast local to the same block, and paired tests for the minimal-force comparison are reported in the text.

Module	Variant	Overall MAE	RMSE	$R^{2}$	Binary F1 (VB ≥ 0.2 mm)	Ordinal Macro-F1
I: Cue construction	OPMST minimal force pair	**0.032**	**0.055**	**0.696**	**0.920**	0.763
	OPMST	0.035	0.059	0.652	0.885	0.772
	MPMST	0.034	0.058	0.669	0.892	**0.786**
II: Score construction	MPMST fixed top-k=6	**0.032**	**0.055**	**0.698**	**0.893**	**0.791**
	MPMST uniform weights	0.034	0.058	0.668	0.892	0.785
	MPMST	0.034	0.058	0.669	0.892	0.786
III: Score transport	OPMST w/o score transport	0.064	0.083	0.324	0.752	0.417
	OPMST	0.035	0.059	0.652	0.885	0.772
	MPMST w/o score transport	0.063	0.080	0.356	0.755	0.425
	MPMST	**0.034**	**0.058**	**0.669**	**0.892**	**0.786**

**Table 6:** Diagnostics for the monotone-score assumption and cue-selection stability over 50 random seeds. AE denotes acoustic emission; CNC internal denotes process signals recorded by the CNC system; KS is the source–target Kolmogorov–Smirnov statistic for raw scores; the unrounded KS means are 0.333235 for MPMST and 0.333271 for OPMST, which both round to 0.333. Jaccard values are reported as CastIron→Stainless/Stainless→CastIron.

Method	Candidate Cue Pool	Target Spearman	Order Violation	Raw Range Coverage	Raw Score KS	Cue Jaccard
MPMST	16 force; 1 vibration; 11 AE; 1 CNC internal	0.887	0.092	0.822	0.333	0.647/0.630
OPMST	47 force-only cues	0.879	0.101	0.831	0.333	0.626/0.625

## Data Availability

The datasets employed in this study are publicly accessible. The intelligent tool condition monitoring dataset for face milling was provided by Mondragon Unibertsitatea and is available at https://hdl.handle.net/20.500.11984/6926 (accessed on 17 March 2026), with DOI https://doi.org/10.48764/3HDP-GF23.

## Abbreviations

The following abbreviations are used in this manuscript:

VB	Flank Wear Width
ADDA	Adversarial Discriminative Domain Adaptation
AE	Acoustic Emission
CDAN	Conditional Domain Adversarial Network
CNC	Computer Numerical Control
CORAL	Correlation Alignment
DANN	Domain-Adversarial Neural Network
FEM-DSAN	Finite Element Method-assisted Deep Subdomain Adaptation Network
ISO	International Organization for Standardization
MAE	Mean Absolute Error
MCD	Maximum Classifier Discrepancy
MPMST	Multi-Physics Monotone Score Transport
MU-TCM	Mondragon Unibertsitatea–Tool Condition Monitoring
OPMST	One-Physics Monotone Score Transport
PG-UDA-PGA	Physics-Guided Unsupervised Domain Adaptation with Physics-Guided Adjustment
PI-GPR	Physics-Informed Gaussian Process Regression
RMSE	Root Mean Square Error
TCA	Transfer Component Analysis
UDA	Unsupervised Domain Adaptation
